# Environmental Presence and Genetic Characteristics of Carbapenemase-Producing *Enterobacteriaceae* from Hospital Sewage and River Water in the Philippines

**DOI:** 10.1128/AEM.01906-19

**Published:** 2020-01-07

**Authors:** Yuki Suzuki, Pearl Joy Nazareno, Ryuichi Nakano, Melisa Mondoy, Akiyo Nakano, Mark Philip Bugayong, Josie Bilar, Mauricio Perez, Emarld Julian Medina, Mariko Saito-Obata, Mayuko Saito, Kazutoshi Nakashima, Hitoshi Oshitani, Hisakazu Yano

**Affiliations:** a Department of Microbiology and Infectious Diseases, Nara Medical University, Kashihara, Japan; b Department of Microbiology, Research Institute for Tropical Medicine, Muntinlupa, Philippines; c Department of Virology, Tohoku University Graduate School of Medicine, Sendai, Miyagi, Japan; d Faculty of Sports and Health Science, Daito Bunka University, Higashi-Matsuyama, Japan; Centers for Disease Control and Prevention

**Keywords:** Carbapenemase-producing *Enterobacteriaceae*, hospital sewage, river water, the Philippines

## Abstract

This study aimed to evaluate the prevalence and genetic characteristics of carbapenemase-producing *Enterobacteriaceae* (CPE) in hospital sewage and river water in the Philippines, which has a typical tropical maritime climate. We collected 83 water samples from 7 hospital sewage and 10 river water sites. CPE were identified using CHROMagar mSuperCARBA, and Gram-negative strains were identified using matrix-assisted laser desorption ionization–time of flight mass spectrometry (MALDI-TOF MS) or 16S rRNA gene sequencing. Resistance genes in *Enterobacteriaceae* strains were identified using PCR and DNA sequencing, and transferability of carbapenemase genes from the CPE was investigated with conjugation experiments. Genotyping was performed using multilocus sequence typing (MLST) for Escherichia coli and Klebsiella pneumoniae. Out of 124 *Enterobacteriaceae* isolates, we identified 51 strains as CPE and divided these into 7 species, 11 E. coli, 14 *Klebsiella* spp., 15 *Enterobacter* spp., and 11 others, including 4 additional species. Conjugation experiments via broth mating and using E. coli J53 revealed that 24 isolates can transfer carbapenemase-encoding plasmids. MLST analysis showed that 6 of 11 E. coli isolates belonged to clonal complex 10 (CC10). Of 11 K. pneumoniae strains, 9 unique sequence types (STs) were identified, including ST147. Five types of carbapenemase genes were identified, with the most prevalent being NDM (*n *= 39), which is epidemic in clinical settings in the Philippines. E. coli CC10 and K. pneumoniae ST147, which are often detected in clinical settings, were the dominant strains. In summary, our results indicate that hospital sewage and river water are contaminated by CPE strains belonging to clinically important clonal groups.

**IMPORTANCE** Carbapenemase-producing *Enterobacteriaceae* (CPE) cause severe health care-associated infections, and their increasing prevalence is a serious concern. Recently, natural ecosystems have been recognized as important reservoirs of antibiotic resistance genes. We investigated the prevalence and genetic characteristics of CPE isolated from the environment (hospital sewage and river water) in the Philippines and found several CPE, including Escherichia coli and other species, with different carbapenemases. The most prevalent carbapenemase gene type was NDM, which is endemic in clinical settings. This study revealed that isolates belonging to carbapenemase-producing E. coli CC10 and K. pneumoniae sequence type 147 (ST147), which are often detected in clinical settings, were dominant in the natural environment. Our work here provides a report on the presence and characteristics of CPE in the environment in the Philippines and demonstrates that both hospital sewage and river water are contaminated by CPE strains belonging to clinically important clonal groups.

## Abstract (precis)

Carbapenemase-producing *Enterobacteriaceae* (CPE) cause severe health care-associated infections, and their increasing prevalence is a serious concern. Recently, natural ecosystems have been recognized as important reservoirs of antibiotic resistance genes. We investigated the prevalence and genetic characteristics of CPE isolated from the environment (hospital sewage and river water) in the Philippines and found several CPE, including Escherichia coli and other species, with different carbapenemases. The most prevalent carbapenemase gene type was NDM, which is endemic in clinical settings. This study revealed that isolates belonging to carbapenemase-producing E. coli CC10 and K. pneumoniae sequence type 147 (ST147), which are often detected in clinical settings, were dominant in the natural environment. Our work here provides a report on the presence and characteristics of CPE in the environment in the Philippines and demonstrates that both hospital sewage and river water are contaminated by CPE strains belonging to clinically important clonal groups.

## INTRODUCTION

Antibiotic resistance, in particular to carbapenems, is a threat to global health. Infections caused by carbapenemase-producing *Enterobacteriaceae* (CPE) have limited treatment options and are associated with high mortality rates (1). CPE cause severe health care-associated infections, and their increasing prevalence is a serious concern (2).

Carbapenemase genotypes originally differed geographically, as the U.S. epidemic was associated with KPC (class A in the Ambler classification) (3), the European epidemic with VIM (class B) and OXA-48-like (class D), and the Asian epidemic with NDM and IMP (class B). The NDM type was first detected in India in tap water and sewage and has now spread to clinical settings worldwide (4, 5). Carbapenemase genes are frequently located on plasmids and mobile genetic elements that can be transmitted between species and often coexist with other classes of antibiotic resistance genes, such as aminoglycosides and fluoroquinolones (6).

The “One Health” approach is a strategic framework for reducing the risk of infectious diseases at the animal-human-environment interface and was officially adopted by international organizations and professional bodies in 2008 (7). Antibiotic resistance has traditionally been viewed as a clinical problem, but within the last decade, natural ecosystems have been recognized as important reservoirs of antibiotic resistance genes (8). CPE have been reported in the natural environment (9), and previous studies have reported the presence of carbapenemase- and/or extended-spectrum β-lactamase-producing *Enterobacteriaceae* in rivers, effluent, and hospital sewage systems worldwide (10–12), highlighting the interrelationship between human infection and water sources (13). It can thus be inferred that the primary origin of these bacteria is humans, from clinical settings and community settings, and that these bacteria are disseminated to the environment.

The CPE epidemic gene type in clinical settings in the Philippines is NDM (NDM-1 and NDM-7) (14, 15); however, the prevalence of CPE in the natural environment and the molecular relatedness between CPE in human settings and CPE in the natural environment are unclear. Therefore, we investigated the prevalence and genetic characteristics of CPE isolated from the environment (hospital sewage and river water) in the Philippines.

## RESULTS AND DISCUSSION

### Identification and characterization of CPE isolates.

We collected 83 water samples from 7 hospital and 10 river sites and then plated them on CHROMagar mSuperCARBA plates to select CPE. High-density colonies grew on all CHROMagar mSuperCARBA plates incubated with the hospital sewage and river water samples. This agar plate inhibits extended spectrum β-lactamase (ESBL)/AmpC producers, and CPE can be selected based on the morphology of red or blue colonies that is characteristic of *Enterobacteriaceae*. In total, 124 *Enterobacteriaceae* isolates were obtained from 83 samples (Table 1), of which 33 were Escherichia coli, 35 *Klebsiella* spp., 28 *Enterobacter* spp., 24 *Citrobacter* spp., 1 Serratia marcescens, 1 Kluyvera ascorbata, 1 Raoultella ornithinolytica, and 1 Providencia stuartii. Among the 124 isolates, 51 were identified as CPE using PCR, and of these, 11 were E. coli, 14 *Klebsiella* spp., 15 *Enterobacter* spp., 8 *Citrobacter* spp., 1 S. marcescens, 1 *K. ascorbata*, and 1 *R. ornithinolytica*. CPE were recovered from five hospital sewage sites (including pretreatment) and four river sites. Of the four hospitals with treatment systems, CPE were detected only from pretreatment samples. Of the 51 CPE, 27 isolates were found in hospital sewage and 24 in river water. For CPE analysis, we removed isolates that have the same antimicrobial susceptibility, resistance genes, and sequence type (ST) patterns to reduce the possibility of having duplicate strains and, finally, obtained 51 CPE. The other 73 isolates were identified as non-CPE as evidenced by negative PCR for carbapenemase; these isolates possibly include duplicate isolates, but further analysis was not performed. Furthermore, we also selected white colonies that were non-*Enterobacteriaceae* and identified them as Acinetobacter spp. and/or *Pseudomonas* spp. using matrix-assisted laser desorption ionization–time of flight mass spectrometry (MALDI-TOF MS). PCR analysis indicated that they possessed some carbapenemases, such as NDM-1, OXA-58, and OXA-72 (16). We would like to further analyze this in detail in the future.

**TABLE 1:** Prevalence of Gram-negative bacteria and carbapenemase-producing *Enterobacteriaceae* (CPE) in the environment

Sampling site (area)	Sewage treatment plant	No. of samples (pretreatment)	No. of *Enterobacteriaceae* isolates grown on CHROMagar mSuperCARBA	No. of isolated CPE (isolates from pretreatment)
Hospital A (Metro Manila)	Yes	2 (12)	33	0 (10)
Hospital B (Metro Manila)	Yes	1 (4)	11	0 (6)
Hospital C (Metro Manila)	Yes	2 (5)	2	0 (2)
Hospital D (Metro Manila)	Yes	1 (3)	3	0 (3)
Hospital E (Metro Manila)	No	3	18	6
Hospital F (Biliran)	No	3	12	0
Hospital G (Leyte)	No	4	16	0
River Q (Metro Manila)	-	5	0	0
River R (Metro Manila)	-	1	2	1
River S (Metro Manila)	-	1	9	9
River T (Metro Manila)	-	3	0	0
River U (Metro Manila)	-	1	1	1
River V (Metro Manila)	-	8	17	13
River W (Rizal)	-	4	0	0
River X (South Cotabato)	-	4	0	0
River Y (Pampanga)	-	4	0	0
River Z (Benguet)	-	12	0	0
Total		83	124	51

Table 2 lists the MICs for antimicrobial agents against the 51 CPE. A total of 44 isolates were nonsusceptible to carbapenems (imipenem or meropenem, ≥2 mg/liter), and 7 were susceptible (4 KPC-2, 2 OXA-48-like, and 1 GES-20 producer). Regarding non-β-lactams, 28 isolates were nonsusceptible to levofloxacin (MIC, ≥4 mg/liter) and 25 were nonsusceptible to gentamicin (MIC, ≥8 mg/liter). A total of 32 isolates with resistance to carbapenems were also resistant to levofloxacin and/or aminoglycosides, and 15 isolates were resistant to carbapenems, fluoroquinolones, and aminoglycosides. The distribution of carbapenemase genes is shown in Table 2. Among the 51 CPE, 39 isolates were positive for NDM (NDM-1, NDM-5, and NDM-7), 7 were positive for KPC (KPC-2), 2 were positive for OXA-48-like (OXA-48 and OXA-181), 2 were positive for GES (two GES-20), and 1 was positive for IMI (IMI-18). In addition, 24 isolates were positive for the CTX-M gene (CTX-M-1G), including CTX-M-15 (22 isolates) and CTX-M-3 (2 isolates). Moreover, NDM producers also carried the CTX-M gene (20/24 isolates). Seven isolates were positive for the 16S rRNA methylase gene RmtC and were highly resistant to aminoglycosides (gentamicin and amikacin MICs, >256 mg/liter); these isolates were all positive for NDM-1. Of the 15 isolates that were resistant to carbapenems, fluoroquinolones, and aminoglycosides, 14 were positive for NDM (4 *Enterobacter* spp., 4 *Citrobacter* spp., 3 E. coli, and 3 *Klebsiella* spp.).

**TABLE 2:** Distribution and characteristics of 51 carbapenemase-producing *Enterobacteriaceae*

Strain no.	Species	Sampling site	Carbapenemase	ESBL^*a*^	16S rRNA methylase	MIC (mg/liter) for^*b*^:					Plasmid incompatibility^*c*^	Transferability^*d*^ (10*^n^*)		ST (CC)^*f*^
						IPM	MEM	LVX	GEN	AMK		Carbapenemase	ESBL	
160	E. coli	Hospital A	NDM-7			2	8	32	32	64	FIA, FIB, X3	nt		448 (448)
222	E. coli	Hospital E	OXA-181	CTX-M-15		0.5	0.5	128	1	4	I1-Iɤ, F, X3	nt	nt	167 (10)
233	E. coli	Hospital E	NDM-7			4	4	16	64	4	FIA, FIB, X3, X4	nt		448 (448)
308	E. coli	River V	OXA-48	CTX-M-15		0.5	0.125	16	1	4	FIA, FIB, F	nt	−8	44 (10)
309	E. coli	River V	NDM-1			4	8	1	1	4	Y, I1-Iɤ, X3	−7		48 (10)
322	E. coli	River V	KPC-2			0.5	0.5	1	8	4	A/C, FIB, Y	nt		206 (206)
331	E. coli	River V	NDM-7			4	2	8	1	8	FIB, F, X3	nt		162 (469)
379	E. coli	River S	KPC-2			1	0.5	16	1	8	X4	−8		617 (10)
438	E. coli	River S	NDM-7	CTX-M-15		4	8	64	2	>256	X3	nt	nt	156 (156)
485	E. coli	River S	NDM-5	CTX-M-15		2	1	32	0.5	8	FIA, FIB, F, X4	nt	nt	167 (10)
536	E. coli	River U	NDM-7	CTX-M-15		8	2	16	0.5	4	FIA, FIB, F, X3	nt	nt	10 (10)
186	K. pneumoniae	Hospital A	KPC-2			2	1	2	128	8	A/C	−7		978
193	K. pneumoniae	Hospital A	KPC-2	CTX-M-15		1	1	2	64	8	A/C	−6	nt	978
270	K. pneumoniae	Hospital B	NDM-1	CTX-M-15	RmtC^*e*^	8	8	4	>256	>256	A/C	−6^*e*^	−6	Novel 1
357	K. pneumoniae	Hospital C	NDM-1	CTX-M-15		4	2	2	16	4	NT	nt	nt	Novel 2
420	K. pneumoniae	Hospital D	NDM-7	CTX-M-15		4	8	4	0.5	2	FIIa, X3	nt	nt	147
221	K. pneumoniae	Hospital E	NDM-1			4	8	64	0.25	4	HI1, X3	nt		37
223	K. pneumoniae	Hospital E	NDM-1	CTX-M-15	RmtC^*e*^	4	4	256	>256	>256	N	−5^*e*^	nt	231
311	K. pneumoniae	River V	NDM-7	CTX-M-15		16	64	64	0.5	4	I1-Iɤ	nt	nt	16
327	K. pneumoniae	River V	NDM-1	CTX-M-15		8	8	32	0.5	8	NT	−6	nt	147
440	K. pneumoniae	River S	KPC-2	CTX-M-3		4	4	16	0.25	4	FIIa	−6	nt	11
484	K. pneumoniae	River S	KPC-2			4	4	2	0.5	2	FII, N, X3	nt		3026
220	Klebsiella oxytoca	Hospital B	NDM-1		RmtC	2	4	2	>256	>256	FIA	nt		
293	K. oxytoca	River V	NDM-7			8	8	64	128	8	A/C, X3	nt		
297	K. oxytoca	River V	GES-20			0.5	0.25	4	0.5	16	L/M	nt		
154	Enterobacter cloacae	Hospital A	NDM-1	CTX-M-15		4	2	1	>256	>256	A/C	nt	nt	
185	E. cloacae	Hospital A	NDM-1	CTX-M-15		4	2	1	128	8	NT	−6	nt	
191	E. cloacae	Hospital A	NDM-1	CTX-M-15		4	2	1	128	4	A/C	−6	nt	
259	E. cloacae	Hospital B	NDM-7			8	4	2	0.5	2	X3	−7		
260	E. cloacae	Hospital B	NDM-1	CTX-M-15	RmtC^*e*^	8	4	1	>256	>256	A/C	−6^*e*^	−4	
264	E. cloacae	Hospital B	NDM-7	CTX-M-3		16	4	1	0.25	2	X3	nt	nt	
267	E. cloacae	Hospital B	NDM-7			4	4	1	0.25	2	X3	−7		
310	E. cloacae	River V	NDM-1	CTX-M-15		4	8	128	64	4	A/C, X3	−7	−4	
460	E. cloacae	River R	IMI-18			32	16	≤0.06	0.25	1	FII	nt		
328	Enterobacter kobei	River V	GES-20			32	4	16	64	8	A/C	nt		
330	Enterobacter tabaci	River V	NDM-1	CTX-M-15	RmtC	64	32	32	>256	>256	A/C	nt	nt	
419	Enterobacter xiangfangensis	Hospital D	NDM-1			2	1	1	0.25	2	A/C	−6		
336	*E. xiangfangensis*	River V	NDM-1	CTX-M-15		8	16	64	128	4	A/C, X3	nt	nt	
377	Enterobacter hormaechei	River S	NDM-1			4	1	0.5	0.5	2	A/C	−6		
376	*Enterobacter* sp.	River S	NDM-1		RmtC	4	16	32	>256	>256	A/C, HI1	−5		
189	Citrobacter freundii	Hospital A	NDM-7	CTX-M-15		8	2	2	0.25	4	A/C, X3	−6	−4	
195	C. freundii	Hospital A	NDM-7	CTX-M-15		8	2	2	0.25	4	X3	−7	−4	
196	C. freundii	Hospital A	NDM-7	CTX-M-15		4	8	16	64	8	A/C, I1-Iɤ, F, X3	nt	nt	
231	C. freundii	Hospital E	KPC-2			1	1	256	0.25	2	A/C	nt		
413	C. freundii	Hospital D	NDM-7			8	4	0.5	0.5	2	HI1, X3	−7		
441	C. freundii	River S	NDM-1			4	2	16	16	4	N	−6		
472	Citrobacter amalonaticus	Hospital A	NDM-1		RmtC^*e*^	8	8	4	>256	>256	N	−6^*e*^		
447	*C. amalonaticus*	River S	NDM-7			8	4	4	128	2	X3	−7		
395	Serratia marcescens	Hospital C	NDM-1			4	1	1	64	8	A/C	−7		
227	Kluyvera ascorbata	Hospital E	NDM-1			1	2	2	0.25	4	X3	nt		
298	Raoultella ornithinolytica	River V	NDM-7			8	16	1	32	4	N, X3	nt		

a ESBL, extended-spectrum β-lactamase.

b Antibiotics: IPM, imipenem; MEM, meropenem; LVX, levofloxacin; GEN, gentamicin; AMK, amikacin.

c NT, nontypeable; underline indicates plasmid incompatibility type of transconjugant.

d nt, not transferred.

e RmtC was also transferred to transconjugants.

f ST, sequence type; CC, clonal complex.

Many CPE, including E. coli and other species, possessed different types of carbapenemase. The most prevalent carbapenemase gene type was NDM (*n *= 39; Table 2); NDM-1 and NDM-7 producers are endemic in clinical settings in the Philippines (14). We found that CPE isolates that are often found in clinical settings were also present in the environment. KPC, GES, and OXA-48 producers, which have not yet been detected in clinical settings in the Philippines (14, 15, 17), were detected in both hospital sewage and river water. Although it is unclear whether these CPE isolates originated from humans or the natural environment, it is possible that they may spread from the natural environment to clinical settings in the future.

### Genotypes of CPE isolated from the environment (E. coli and K. pneumoniae).

Various sequence types (STs) were identified from multilocus sequence typing (MLST) analysis of E. coli and Klebsiella pneumoniae (Table 2). Six E. coli isolates belonged to clonal complex 10 (CC10; ST10, ST44, ST48, ST167, and ST617). Moreover, 9 STs were identified, including 2 novel STs, in 11 K. pneumoniae isolates (2 ST147, 2 ST978, 1 ST11, 1 ST16, 1 ST37, 1 ST231, and 1 ST3026).

Carbapenemase-producing E. coli isolates were divided into nine unique MLST types based on MLST analysis. E. coli belonging to ST131 is a major ST of ESBLs and/or carbapenemase-producing isolates in humans (18, 19); however, it was not detected in this study. Six isolates belonging to CC10 contained the carbapenemase gene types NDM (NDM-1, NDM-5, and NDM-7), KPC-2, and OXA-48-like (OXA-48 and OXA-181). A study conducted in the Netherlands found that ESBL-producing E. coli belonging to CC10 is present in a broad range of hosts, including humans, animals, and environmental surface water, with almost the same frequency (10%) (20). Moreover, CC10 has also been reported in the Danube River (Europe) (21), the Yamato River (Japan) (22), and sewage in Pakistan (23). A possible explanation is that CC10 circulates easily among different hosts and contains different types of carbapenemase genes. Therefore, the presence of carbapenemase-producing E. coli belonging to CC10 in the environment is a concern, because it can spread among humans, animals, and the environment.

Of the 11 K. pneumoniae strains identified, 2 isolates belonged to ST147 (1 NDM-1 producer and 1 NDM-7 producer) and 1 belonged to ST11 (a KPC-2 producer). In clinical settings worldwide, K. pneumoniae strains that belong to ST11, ST14, ST101, ST147, and ST258 are major carbapenemase-producing clones (24). K. pneumoniae strains that produce NDM-1 belonging to ST147 and KPC-2 belonging to ST11 are predominant in Germany and China in clinical settings, respectively (25, 26). We found that CPE isolated from clinical settings were also present in the natural environment, suggesting that CPE isolated from humans and the environment may be connected.

We found carbapenemase-producing E. coli belonging to CC10 and K. pneumoniae belonging to ST147 and ST11 in hospital sewage as well as in river water. It is probable that some hospital and domestic sewage is directly discharged into municipal sewage, and thus CPE were detected in river water. The Global Water Intelligence report states that the sewage system coverage rate is 31.2% in the Philippines, which is similar to that in other Southeast Asian countries (30 to 40%) (27); meanwhile, the coverage in Europe and North America is 70 to 80% (27). A previous study reported that CPE are spread throughout a community area by direct discharge (11); therefore, a broad sewage system coverage rate may prevent human contact with CPE-contaminated water. Increasing the sewage system coverage rate is expected to reduce the presence of CPE in the environment; however, a more in-depth understanding is needed because there are various factors at play, such as climate and regionality. Given the observed prevalence of CPE in river water and hospital sewage, transmission to humans through water contact is a realistic possibility. The probability of transmission depends upon the function and frequency of contact between humans and contaminated water bodies, e.g., whether for recreational activity or irrigation. In this study, CPE were detected only in pretreatment sewage, and not in posttreatment sewage, of four hospitals (hospitals A to D in Table 1). These hospitals treat sewage via aeration, chlorine treatment, and filtration. We theorized that CPE were not detected in posttreatment sewage due to the treatment process, which reduces the number of sewage bacteria. These findings highlight the need for constant monitoring of hospital sewage for antibiotic-resistant bacteria and for efficient sewage treatment plants in health care settings as part of biosecurity programs. Moreover, the findings support the importance and urgency of action needed to reduce environmental contamination by CPE.

### Characteristics of carbapenemase-encoding plasmids and their transferability.

Replicon typing revealed variability in the types of carbapenemase-encoding plasmids encountered in terms of incompatibility groups. Of the 51 isolates, 23 and 19 possessed X3 (circulating among *Enterobacteriaceae*) and A/C (broad-host-range) replicon regions, respectively, including E. coli, *Enterobacter* spp., and *Klebsiella* spp., whereas 3 isolates possessed nontypeable plasmids. Of the 23 IncX3-possessing isolates, 21 were NDM producers. All CPE isolates were tested for transferability of carbapenemase gene determinants by conjugation with E. coli J53, and transconjugants containing carbapenemase-encoding plasmids were obtained from 24 (47.0%) isolates, with an average transfer frequency of 3.1 × 10^−6^ (range, 10^−5^ to 10^−8^). Of the 24 transconjugants, 20 were positive for NDM and 4 were positive for KPC. There was no significant difference in transfer frequency between NDM and KPC producers.

Among the 20 transferable NDM producers, the most prevalent plasmid incompatibility types were IncA/C (*n* = 9) and IncX3 (*n* = 8), which have a broad host range (28). Of the NDM-positive transconjugants, IncX3 and A/C plasmids were obtained from 7 and 1 isolate, respectively, while the remaining 12 transconjugants had nontypeable plasmids (Table 2). Although the transfer frequency was not high (average transfer frequency, 10^−6^), it is possible that the NDM-encoded plasmids are transferred to other species and strains. Indeed, IncX3 plasmids carrying various carbapenemases, such as NDM and KPC, were isolated from both clinical settings and sewage in the United Arab Emirates (UAE) (29), Myanmar (30), and China (31). Therefore, monitoring the prevalence of IncX3 plasmids in clinical settings and in sewage is necessary. Regarding the ESBL genes, conjugation experiments revealed that 6 of 24 CTX-M-1G-positive isolates were able to transfer their CTX-M-encoded plasmids to E. coli J53 (Table 2). It is possible that the carbapenemase and CTX-M genes are located on nontransferable plasmids or chromosomes of isolates that were not able to transfer both resistance genes. A previous study reported the presence of CTX-M and carbapenemase genes on chromosomes, raising concerns that these strains may disseminate worldwide (32–34). Nevertheless, it is not clear whether resistant genes that are not transferred are located on plasmids or chromosomes, and thus further analysis of the genome via next-generation sequencing is warranted.

In addition, we identified species rarely reported in clinical settings, such as *K. ascorbata* and *R. ornithinolytica*, which produce NDM. This suggests that carbapenemase-encoding genes are spreading to nonconventional organisms and may be more widespread in the environment than previously thought.

### Conclusion.

Our study reports the presence and genetic characteristics of CPE in the environment (hospital sewage and river water) in the Philippines. Various species produced different carbapenemases, with the most prevalent gene type being NDM, which is epidemic in clinical settings. We found that isolates belonging to carbapenemase-producing E. coli CC10 and K. pneumoniae ST147, which are also often detected in clinical settings, were dominant in the natural environment. Further studies are warranted for investigating the epidemiological links between isolates from the natural environment and humans.

## MATERIALS AND METHODS

### Collection of environmental samples.

**FIG 1:**
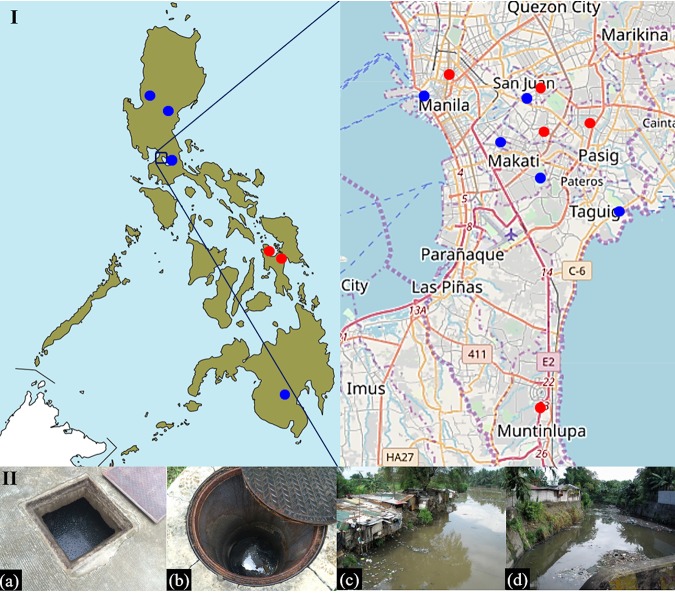
Map of the study area. I. Red dots indicate hospital sewage sites, and blue dots indicate river water sites. Map templates were taken from http://www.freemap.jp/ (left) and https://www.openstreetmap.org/ (right). II. Sampling sites. (a and b) Hospital sewage; (c and d) river.

We collected water samples from 7 hospital sewage and 10 river sites between August 2016 and August 2018. The study area included Metropolitan Manila, Benguet, South Cotabato, Pampanga, Rizal, Leyte, and Biliran, Philippines, which have a typical tropical maritime climate (Table 1, Fig. 1). For hospital sewage, pretreatment samples were collected from seven hospitals. Moreover, four of the seven hospitals had sewage treatment systems, and thus posttreatment samples were also collected.

### CPE selection and species identification.

To select for CPE, 100 μl of each water sample was plated on CHROMagar mSuperCARBA (Kanto Chemical, Tokyo, Japan) and incubated at 35°C for 24 to 48 h. We selected 20 to 50 colonies that differed in form and color and gave priority to red and blue colonies with morphology characteristic of *Enterobacteriaceae*, according to the manufacturer’s instructions. Primary identification was conducted with MALDI-TOF MS using a Vitek MS system (bioMérieux, Marcy-l’Étoile, France). Isolates that could not be identified using MALDI-TOF MS were identified using 16S rRNA gene sequencing (35).

### Antimicrobial susceptibility testing.

The antimicrobial susceptibility of various antimicrobial agents was determined using the agar dilution method (36), and quality control was performed using E. coli ATCC 25922. MICs were interpreted according to the breakpoints defined by the Clinical and Laboratory Standards Institute (37).

### Detection of antimicrobial resistance genes.

PCR was performed for all isolates from the environmental samples using AmpliTaq Gold 360 master mix (Thermo Fisher Scientific, Waltham, MA) to detect the carbapenemase genes *bla*_IMP_, *bla*_VIM_, *bla*_KPC_, *bla*_OXA-48-like_, *bla*_NDM_, *bla*_GES_, *bla*_IMI_, and *bla*_SME_ (38–44). Carbapenemase-positive isolates were also tested for other resistance genes using PCR, including CTX-M-type ESBL (*bla*_CTX-M-1Group_, *bla*_CTX-M-2G_, *bla*_CTX-M-9G_, and *bla*_CTX-M-8/25G_) and 16S rRNA methylase genes (*armA*, *rmtA*, *rmtB*, *rmtC*, *rmtD*, and *npmA*) (45–47).

DNA sequencing was conducted using BigDye Terminator version 3.1 (Applied Biosystems, Foster City, CA) and an ABI3730xl analyzer (Applied Biosystems). BLAST version 1.12 (https://blast.ncbi.nlm.nih.gov/Blast.cgi) was used to process the sequencing data and to identify genes.

### Plasmid characterization and conjugation experiments.

Plasmid incompatibility groups were identified using the PCR-based replicon-typing method (48, 49). Conjugation experiments were conducted with the broth mating method using CPE isolates as the donor and sodium azide-resistant E. coli J53 as the recipient as previously described (50). Exponential-phase Luria-Bertani broth cultures of donor strains and recipient E. coli J53 were mixed at a ratio of 1:1 (by volume); these mating mixtures were incubated overnight at 35°C. Transconjugants were selected on Luria-Bertani agar plates containing cefpodoxime (8 μg/ml) and sodium azide (100 μg/ml).

### MLST.

MLST was performed using Achtman’s (51) and Institut Pasteur’s (52) schemes for E. coli and K. pneumoniae isolates, respectively. Housekeeping genes in E. coli (*adk*, *fumC*, *gyrB*, *icd*, *mdh*, *purA*, and *recA*) and K. pneumoniae (*rpoB*, *phoE*, *infB*, *gapA*, *mdh*, *pgi*, and *tonB*) were sequenced. DNA sequence variations were analyzed using an MLST database for E. coli and K. pneumoniae (https://pubmlst.org/bigsdb?db=pubmlst_mlst_seqdef) to determine STs.
